# Development and external validation of a prognostic model for occult atrial fibrillation in patients with ischemic stroke

**DOI:** 10.3389/fneur.2022.1034350

**Published:** 2023-01-18

**Authors:** Xuan Wang, Longyan Meng, Yanxin Zhao, Xueyuan Liu

**Affiliations:** Department of Neurology, Shanghai Tenth People's Hospital, Tongji University School of Medicine, Shanghai, China

**Keywords:** acute ischemic stroke, occult atrial fibrillation, nomogram, secondary prevention, cardioembolism

## Abstract

**Objective:**

Currently, the risk of occult atrial fibrillation (AF) could not be predicted in patients with acute ischemic stroke (AIS) using a simple scoring system. Therefore, in this study, we developed and externally validated a nomogram to predict occult AF in patients with AIS.

**Methods:**

In this study, we prospectively conducted a development cohort study with data collected at our stroke center from July 2017 to February 2018, and an external validation cohort from March 2019 to December 2019.

**Results:**

Follow-up data were collected from 177 participants (56.5% older than 65 years, 29.4% female) for generating the nomogram model. Multivariate logistic regression analysis was performed with AF as the dependent variable indicated that age >65 years, heart rate >100, C-reactive protein (CRP), N-terminal pro-B-type natriuretic peptide (NT-proBNP) >270, hemorrhagic transformation (HT) as independent variables for predicting the development of AF, and a nomogram was generated based on these factors. The area under the receiver operating characteristic curve (AUC-ROC) for the model was 0.937, the C-index was 0.926, and the AUC-ROC for the validation cohort was 0.913.

**Conclusion:**

To our knowledge, this is the first nomogram developed and externally validated in a stroke center cohort for individualized prediction of risk of developing AIS in patients with occult AF. This nomogram could provide valuable information for the screening of occult AF after a stroke.

## Introduction

Atrial fibrillation (AF) increases the risk of stroke by four to five times (1), and according to early estimates of the prevalence of AF, 15%−20% of strokes are caused by AF (2). AF-related stroke generally has a poor clinical outcome, although anticoagulation is largely preventable (64% lower risk of stroke and 25% lower mortality) (3). Currently, 12 million ischemic stroke cases are reported each year, and one in four strokes and half of transient ischemic attacks (TIAs) have no established cause after standard diagnostic testing and are marked as “cryptogenic” (4). Undiagnosed AF is believed as the cause of many cryptogenic strokes (CSs); however, anticoagulation is not indicated unless AF is proven. Because patients with AF have an increased risk of recurrent stroke, improved detection and treatment strategies for AF are expected to reduce the burden of recurrent stroke. The detection of AF has a substantial impact on the treatment decisions of patients with ischemic stroke. Subclinical AF is one of the pathogenesis of embolic stroke of unknown origin (ESUS) (5). According to statistics, AF accounts for approximately 8%−15% of ESUS cases (5, 6); even with direct oral anticoagulation, empirical anticoagulation is no better than aspirin in preventing recurrent strokes without documented AF (7, 8). Therefore, in the absence of documented AF, aspirin remains the standard antithrombotic therapy (9). Nevertheless, in patients with ESUS, the annual recurrence rate with standard antithrombotic therapy remains as high as 5% (10). Therefore, early etiological classification of patients with acute ischemic stroke (AIS) can select a reasonable secondary prevention program for the patients. However, the screening of some types of AF is not easy, and accurate etiological classification is highly dependent on the results of complete auxiliary examinations. Occult AF is often transient and asymptomatic, so there are challenges in clinical screening work (11, 12). The Chinese National Stroke Registry data showed that the proportion of stroke or TIA patients with AF in China is only 6.7% (13), while a large-scale registration study conducted in Europe showed that the proportion is 38% (14); thus, it can be seen that the detection rate of AF in China is significantly lower than that in Europe. Similarly, in terms of secondary prevention in patients with AIS complicated by AF, the situation in China is also significantly worse than that in developed countries. Compared with foreign countries, in China, the diagnosis rate of AF is low, and the missed diagnosis rate is high. Screening for this risk factor makes the diagnosis of occult AF difficult. If a reliable screening method can be found to preliminarily predict the possibility of cerebral embolism caused by occult AF in patients with AIS, it can guide further targeted examinations to determine whether it is occult AF, which will greatly improve the diagnosis rate of occult AF. At present, there is no well-recognized and very effective primary screening method for differential diagnosis. Therefore, the importance of screening AF is obvious. Screening high-risk patients, examining them more accurately, and improving the etiological diagnosis of occult AF will not only affect the direction of treatment but also guide the choice of stroke prevention programs in this group.

## Methods

### Study design and patient enrollment

This is a longitudinal study aimed at developing and validating prediction models. A total of 401 patients with AIS who were hospitalized in the Stroke Center of the Tenth People's Hospital affiliated with Tongji University in Shanghai from July 2017 to February 2019 were consecutively recruited. Participants met the following criteria: (1) 18 years of age or older and (2) diagnosed with AIS based on diffusion-weighted magnetic resonance imaging (MRI) within 1 week, and patients were excluded if key data were missing. The validation cohort included 65 stroke subjects without previous documented AF who were admitted from the same stroke center between March 2019 and December 2019. The inclusion/exclusion criteria for the development cohort also apply to the external validation cohort. AF was defined as a prior history or diagnosis of AF after hospital discharge. A history of a previous stroke was defined as a history of a previous ischemic stroke or TIA. According to our stroke center clinical reference value range, a high heart rate on admission was defined as a heart rate >100 beats/min, high TnT was defined as a TnT >0.014 ng/ml, and high NT-proBNP was defined as an NT-proBNP >270 pg/ml. Age and AF-related ROC curves showed an optimum sensitivity and specificity trade-off at age 65 years, so patients were grouped according to the optimal age cutoff of 65 years, the elderly patients are defined as those of age >65 years. HT is defined as the first head CT/MRI after AIS without bleeding, and the second head CT/MRI examination hemorrhagic infarction can be identified by the finding of intracranial hemorrhage or by the first CT/MRI findings. Multiple ischemic lesions in MRI are defined as infarcts caused by the occlusion of two or more different cerebral vessels in the blood supply system. Shanghai Tenth People's Hospital Ethics Committee approved this study, and all participants and their carers provided written informed permission.

### Data collection

Demographic data such as sex and age, current medical history, vascular risk factors, and the National Institutes of Health Stroke Scale (NIHSS) admission scores were collected. Laboratory test data such as routine blood count, blood biochemistry, and coagulation function were collected from all patients based on medical records during hospitalization. Relevant imaging data, including brain imaging, routine electrocardiogram, transthoracic echocardiography, and 24-h Holter heart rate test were also collected. All patients included in the external examination of the model were examined by a 24-h dynamic electrocardiogram. Based on whether AF was present or not, patients were divided into AF and non-AF groups.

### Statistical analysis

Continuous quantitative variables are shown as mean and standard deviation, while categorical variables are shown as frequencies and ratios. First, the normal distribution of quantitative variables was determined using the Kolmogorov–Smirnov test. Student's *t*-test was then used to compare normally distributed quantitative data, while the chi-square test was employed to compare qualitative variables. The Mann–Whitney *U*-test was used to compare non-normally distributed variables. Based on the odds ratio (OR) and 95% confidence interval (CI), we conducted a logistic regression analysis with a probability of entry set to 0.05 and a probability of removal set to 0.10. To build the prediction model, variables with a *p*-value of < 0.05 in the multivariate analysis were incorporated into the R language. AUC-ROC curves were used to calculate the predictive accuracy of nomogram models to differentiate patients with AF. Then, we used the bootstrap method (1,000 resamplings) for internal verification and to calculate a revised C-index, which is equivalent to a range of 0.5–1.0 for AUC-ROC. The higher the score, the more accurate the prediction. Models built from the development cohort were then applied to an external validation cohort, and performance was evaluated by AUC-ROC. Calibration of the risk prediction model in the development cohort was performed by comparing the observed AF probabilities according to the nomogram-based total score with the nomogram-based predicted probabilities and assessed using the Hosmer–Lemeshow test whether event rates observed in patients with AIS match expected rates. In all cases, a *P*-value of < 0.05 is considered statistically significant. We used R software version 3.6.2 (2019 R Foundation for Statistical Computing platform), SPSS 24 (IBM Corporation, New York, USA), and GraphPad Prism 7 (GraphPad Software, La Jolla, CA, USA) for the analysis.

## Results

### Development cohort characteristics

Of all 401 patients, 224 were excluded from data analysis due to incomplete examination and lack of baseline data; finally, 177 patients were analyzed. Compared with included patients, those excluded due to the lack of baseline data had no significant difference in terms of age >65 (59.8 vs. 56.5%), female gender (30.8 vs. 29.4%), NIHSS on admission (3.53 ± 3.88 vs. 3.48 ± 3.84), hypertension (66.1 vs. 74%), diabetes mellitus (37.5 vs. 40.1%), AF (29.0 vs. 37.9%), and *p* >0.05 for all (Supplementary Table S1). As shown in Table 1, the mean age was 68.7 ± 12.1 years, 52 patients (29.3%) were women, and the mean NIHSS score at presentation was 3.5 ± 3.1. A total of 67 patients (49 were previously diagnosed, and 18 were diagnosed after discharge) were identified as having AF. Univariate analysis found that age >65, sex, NIHSS score, hypertension, history of the previous stroke, heart rate >100, aortic sinus diameter, left atrial diameter, CRP, platelet count, D-D dimer, triglyceride, high-density lipoprotein, TnT > 0.014, NT-proBNP > 270, HT, and multiple ischemic lesions in MRI were significantly different between AF and non-AF groups (*P* < 0.05 for all). Multivariate logistic regression was conducted with these factors.

**Table 1:** Baseline and procedural characteristics.

**Characteristics**	**Non-AF (*n =* 110)**	**AF (*n =* 67)**	***P*-values**
Age>65	44 (40%)	56 (83.6%)	0.0001
Female, n (%)	25 (22.7%)	27 (40.3%)	0.013
NIHSS on admission, means (SD)	2.51 (2.79)	5.07 (4.72)	0.0001
**Vascular risk factors, n (%)**			
Hypertension	87 (79.1%)	44 (65.7%)	0.048
Diabetes mellitus	48 (43.6%)	23 (34.3%)	0.220
Previous stroke	16 (14.5%)	19 (28.4%)	0.025
Heart rate	74.25 (13.54)	87.48 (24.28)	0.0001
Heart rate >100	21 (19.1%)	23 (34.3%)	0.023
Aortic sinus inner diameter	35.05 (4.31)	33.70 (3.82)	0.037
Left atrial diameter	37.41 (4.25)	42.73 (6.95)	0.0001
**Laboratory findings**			
C-reactive protein, means (SD)	5.80 (8.53)	15.57 (24.09)	0.002
WBC, means (SD)	7.50 (7.72)	7.79 (3.30)	0.724
Platelet count (× 109/L)	212.62 (50.97)	196.46 (53.54)	0.046
PT, means (SD)	11.29 (1.02)	11.52 (1.11)	0.149
INR, means (SD)	0.97 (0.09)	1.39 (2.24)	0.131
APTT, means (SD)	28.09 (3.26)	28.95 (6.62)	0.323
Fibrinogen (g/L)	3.02 (0.69)	3.15 (0.75)	0.239
D-Dimer, means (SD)	0.78 (1.16)	1.52 (2.59)	0.025
Triglyceride (mmol/L)	1.61 (1.00)	1.22 (0.62)	0.004
Total cholesterol (mmol/L)	4.16 (1.16)	4.02 (1.08)	0.429
HDL-C (mmol/L)	1.05 (0.23)	1.13 (0.31)	0.025
LDL-C (mmol/L)	2.62 (0.97)	2.37 (0.97)	0.102
TnT (ng/mL)	0.02 (0.03)	0.03 (0.04)	0.024
TnT > 0.014, n (%)	35 (31.8%)	44 (65.7%)	0.0001
NT-proBNP (pg/ml)	278.18 (710.79)	1,811.60 (1,952.17)	0.0001
NT-proBNP > 270, n (%)	21 (19.1%)	61 (91%)	0.0001
Hemorrhagic transformation, n (%)	2 (1.8%)	12 (17.9%)	0.0001
Multiple ischemic lesions in MRI, n (%)	51 (46.4%)	42 (62.7%)	0.035

NIHSS, National Institute of Health Stroke; CRP, C-reactive protein; HDL-C, high-density lipoprotein cholesterol; LDL-C, low-density lipoprotein cholesterol; TnT, troponin T; NT-proBNP, N-terminal pro-B-type natriuretic peptide.

### The development of an individualized prediction model

**Figure 1:**
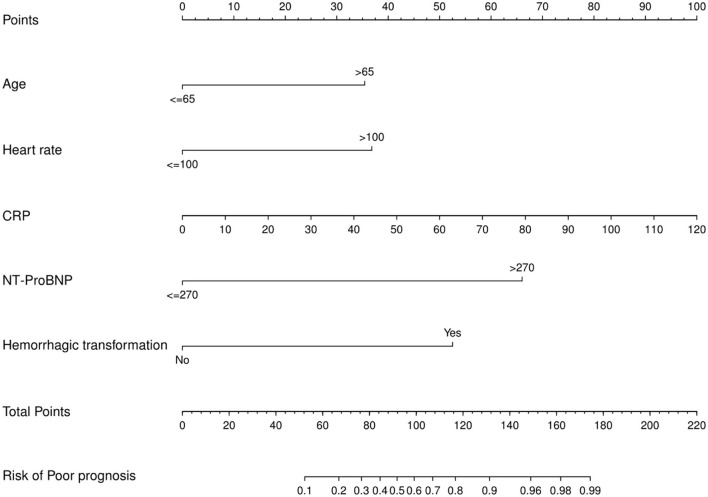
Nomogram for predicting the probability of AF in stroke.

**Figure 2:**
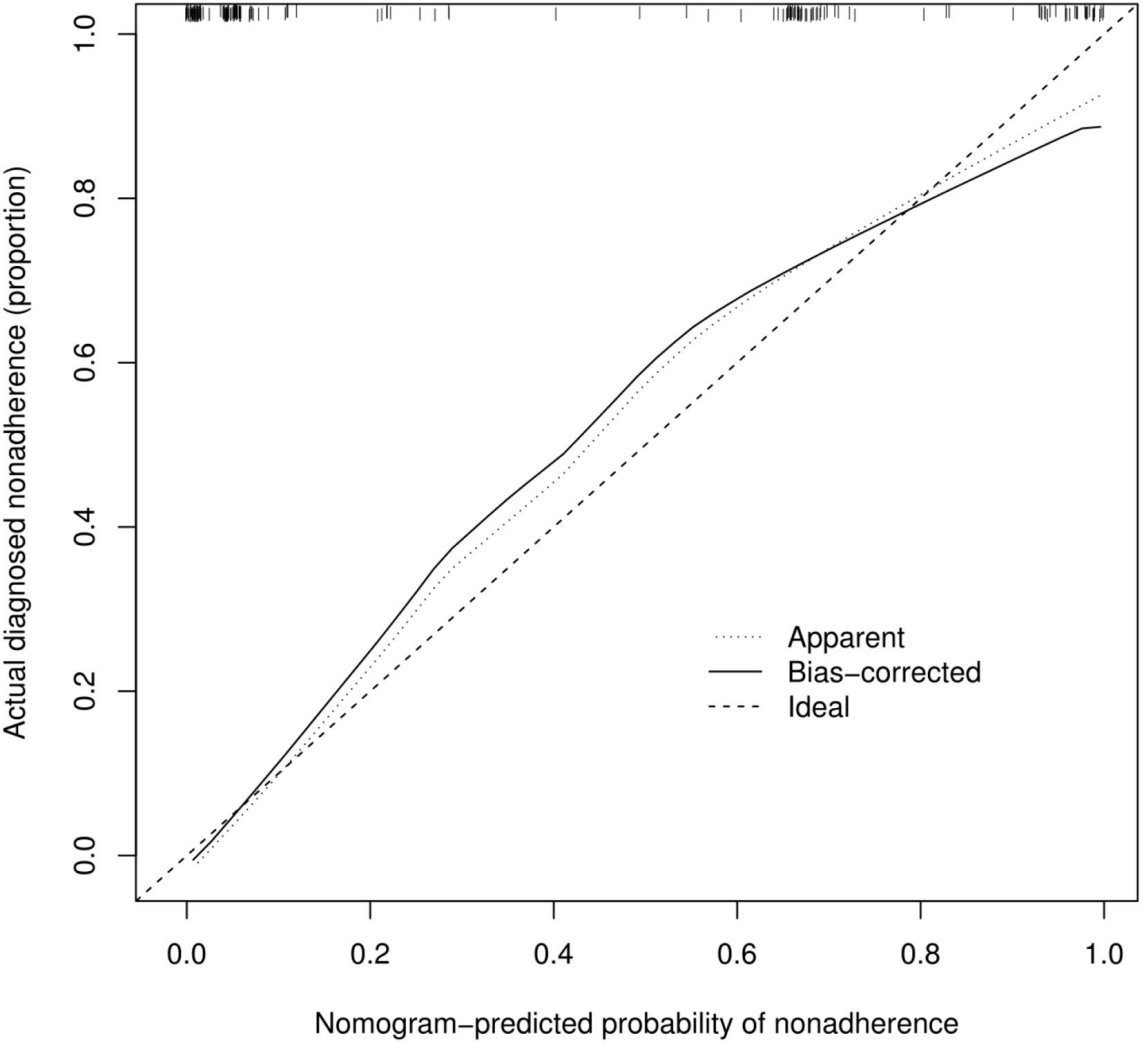
Predictive model based on logistic analysis for early diagnosis of AF in stroke in development and validation cohorts.

In binary logistic analysis, sex, NIHSS score, hypertension, history of previous stroke, heart rate, aortic sinus diameter, left atrial diameter, platelet count, D-D dimer, triglyceride, high-density lipoprotein, TnT > 0.014, and multiple ischemic lesions in MRI were excluded because they were not statistically significant. As shown in Table 2, for the development of the model, five potential predictors were generated by multivariate logistic regression (LR method): age > 65 (OR, 4.95, 95% CI, 1.18–20.76; *p* = 0.029), heart rate > 100 (OR, 8.04, 95% CI, 1.99–32.48; *p* = 0.003), CRP (OR, 1.06, 95% CI, 1.00–1.11; *p* = 0.042), NT-proBNP > 270 (OR, 20.01, 95% CI, 4.27–93.74; *p* = 0.0001), and HT (OR, 32.24, 95% CI, 2.47–420.79; *p* = 0.008). The nomogram was used to build a prediction model. The point values of each factor used to calculate the total score are summarized as shown in Figure 1. The area under the ROC curve for the developed validation was 0.937, with a sensitivity of 95.5% and a specificity of 85.5% (Figure 2).

**Table 2:** Multivariate analysis for predictors of AF-related stroke (*n* = 177).

**Variable**	**OR**	**95%CI**	***P*-values**
Age>65	4.95	1.18–20.76	0.029
Female	3.41	0.81–14.32	0.094
NIHSS on admission	1.05	0.88–1.25	0.574
Hypertension	0.30	0.08–1.16	0.080
Previous stroke	1.48	0.39–5.59	0.563
Heart rate>100	8.04	1.99–32.48	0.003
Aortic sinus inner diameter	1.00	0.84–1.18	0.975
Left atrial diameter	1.11	0.99–1.25	0.078
CPR	1.06	1.00–1.11	0.042
Platelet count	1.01	1.00–1.02	0.173
D-Dimer	0.92	0.55–1.53	0.743
Triglyceride	0.75	0.32–1.78	0.516
HDL-C	1.28	0.17–9.40	0.810
TnT > 0.014	3.02	0.74–12.29	0.124
Pro-BNP > 270	20.01	4.27–93.74	0.0001
Multiple ischemic lesions in MRI	1.06	0.32–3.46	0.924
Hemorrhagic transformation	32.24	2.47–420.79	0.008

NIHSS, National Institute of Health Stroke; CRP, C-reactive protein; HDL-C, high-density lipoprotein cholesterol; TnT, troponin T; NT-proBNP, N-terminal pro-B-type natriuretic peptide.

### External validation and apparent performance of the occult AF risk nomogram

**Figure 3:**
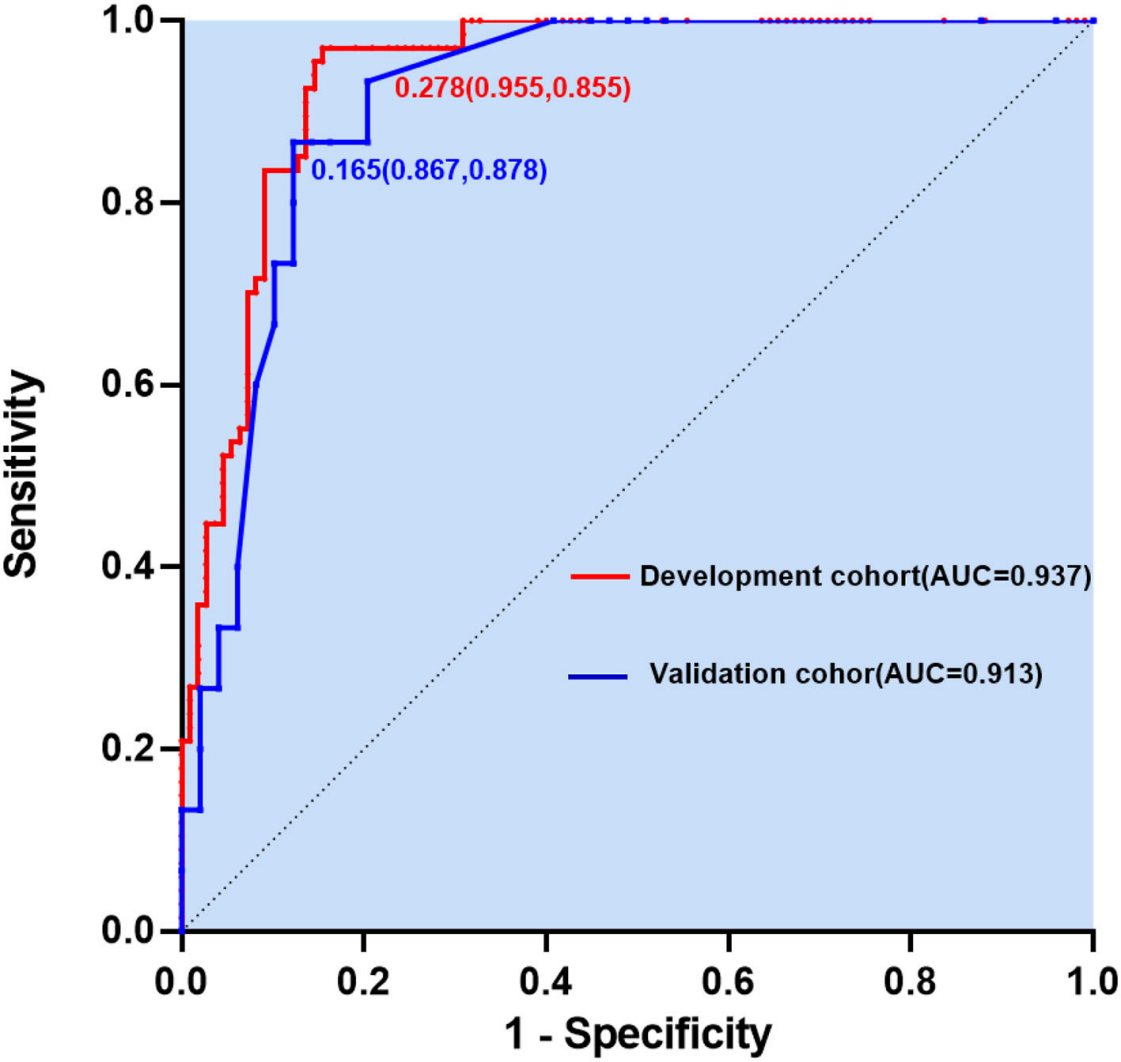
Calibration curve for the nomogram-predicted probability of AF in stroke.

According to Figure 3, the calibration curve of the nomogram for predicting occult AF in patients with AIS in this cohort showed good agreement. The resulting estimated AUC value of 1,000 bootstrap samples is 0.937, indicating that the model has good discrimination. The validation cohort's area under the ROC curve was 0.913, as illustrated in Figure 2, with a sensitivity of 86.7% and a specificity of 87.8%. Therefore, in both the internal and external variations, our nomogram had excellent prediction performance, suggesting the nomogram based on the five existing risk factors has a high degree of generalizability.

## Discussion

In this study, the risk factors associated with AF in patients with stroke were identified using logistic regression analysis. In addition, we developed and externally validated a screening model for predicting occult AF in patients with stroke to facilitate the screening of high-risk patients with occult AF.

Studies have shown that cryptogenic stroke (CS) accounts for about 35% of all ischemic strokes (15), and the literature shows that approximately 67% of patients initially diagnosed with CS are found to have evidence of underlying cardioembolic stroke after a period of follow-up (16). Meanwhile, the main cause of cardioembolic stroke is AF. Compared with paroxysmal AF, persistent AF and permanent AF are easier to diagnose. We have observed from clinical work that paroxysmal AF is more likely to be missed during hospitalization. Many studies have reported that the risk of AIS due to paroxysmal AF is not significantly different from persistent AF and permanent AF (17). In addition, ECG monitoring for patients with AIS lasting >48 h can increase the detection rate of AF by 17.5–21.3% (18). However, the detection of AF, especially paroxysmal AF, remains limited due to the uneven distribution of medical resources.

Hence, we established a nomogram based on age, heart rate, CRP, NT-proBNP, and HT to accurately predict the probability of occult AF in patients with AIS. By combining readily available clinical information with this model, clinicians can predict occult AF in patients with AIS rapidly and personally.

Both our findings and earlier research indicate that the association of AF with stroke increases with age (14, 19). In clinical work, the CHA2DS2-VASc score is widely used clinically to evaluate whether patients with a definite diagnosis of AF need anticoagulation, and factors such as age >65 years in the scoring system are considered to be relatively higher risk. Apart from advanced age, some studies have shown that NT-proBNP is also an independent predictor of paroxysmal AF (20). NT-proBNP has the advantages of a long half-life and good stability and is more suitable for clinical use. Serum NT-proBNP levels were considerably greater in patients with AF than in sinus rhythm controls (21). Lucie Garnie et al. (22) suggested that post-stroke AF was independently predicted by NT-proBNP. Our data further demonstrate the close association of NT-proBNP with AF in patients with AIS. The STAF scoring system developed by Suissa et al. (19) found that higher NIHSS scores, left atrial enlargement, asymptomatic extracranial stenosis (*I* > 50%), or cavitary infarction syndrome were associated with AF in patients with stroke. However, a prospective study from China showed that the STAF score has only moderate sensitivity and specificity in detecting AF in patients with stroke, with a relatively limited ability to predict AF, especially paroxysmal and new-onset AF (23).

In addition to the aforementioned factors, higher CRP at admission was also a strong modifiable predictor of AF. A previous study found that atrial inflammation is an important factor in the pathogenesis of AF. In long-term persistent/permanent AF, inflammatory infiltration and CRP levels in blood were significantly increased, and it was found that the degree of atrial inflammation was closely related to CRP blood levels (24), and the significance of CRP in predicting occult AF in individuals with AIS is further supported by our investigation. In our model, the strongest predictor of AF was HT. In fact, few studies have explored the relationship between HT and stroke complicated with AF, whereas a recent article published in *Stroke* pointed out that AF is associated with cerebral parenchymal hematoma and symptomatic intracranial hemorrhage after AIS (25). In multivariate analysis, wide 95% confidence intervals of HT and NT-proBNP suggest that these patients have a lower overall incidence of events and a higher incidence of AF. The blood biochemical, ultrasonography, imaging, and other indicators used for screening AIS complicated with occult AF have the characteristics of non-invasiveness, simple detection operation, and low price, which are more convenient for clinical application and promotion, and provide a new alternative diagnostic method for the diagnosis of cardiogenic embolism. Therefore, for high-risk groups, more accurate examinations such as long-term electrocardiography can be carried out in a targeted manner, observe the condition closely, repeat multiple examinations, increase the detection rate of AF, choose a more appropriate antithrombotic plan, thereby reducing the risk of cardioembolic stroke and death.

There are several limitations that need to be emphasized. First, in the developed validation, we included all patients with stroke in the study, not just those with cryptogenic stroke. We did this because the sample size is small and the number of ESUS is relatively small, and it is difficult to establish a validation model in the development cohort if we focus only on patients with ESUS, but external validation of the predictive model for occult AF in the ESUS patient cohort further confirmed the reliability of the model, which may compensate for the shortcomings introduced by our experimental design. Second, due to the limited conditions and the number of days the patients were in the hospital, we only performed 24-h dynamic electrocardiogram and not long-term ECG monitoring for all patients in the validation cohort, which leaves our prediction model somewhat deficient in detecting occult atrial AF. However, due to the limited conditions at our stroke center and the number of days the patients were in the hospital, it was difficult for us to complete long-term ECG monitoring such as 14 vs. 30 days of external cardiac monitoring during hospitalization, and the long-term ECG follow-up will be refined as much as possible in future clinical work. Third, compared with included patients, those excluded due to lack of baseline data showed a trend toward lower hypertension and AF (both *p* < 0.10), and there may be an inadvertent selection bias in place. Finally, this study is a single-center study with the problems of a single population and a small sample size, which may cause natural bias. If possible, external validation should be considered in a multicenter cohort with a large number of patients.

## Conclusion

This study provides a simple screening model for occult AF in acute stroke patients. This externally validated nomogram provides clinicians with a new tool that is more useful than traditional patient consultation risk scores because it makes it easy and fast to visualize each stroke survivor's risk.

## Data availability statement

The original contributions presented in the study are included in the article/Supplementary material, further inquiries can be directed to the corresponding authors.

## Ethics statement

The studies involving human participants were reviewed and approved by Shanghai Tenth People's Hospital, Tongji University School of Medicine, 301 Middle Yanchang Road, Shanghai 200072, China. The patients/participants provided their written informed consent to participate in this study. Written informed consent was obtained from the individual(s) for the publication of any potentially identifiable images or data included in this article.

## Author contributions

YZ and XL conceived and designed the study. XW collected and analyzed the data. LM and XW drafted the article. All authors read and approved the version of the final manuscript.
