# Supplementary material for: Development and external validation of a prognostic model for occult atrial fibrillation in patients with ischemic stroke
Source: Front Neurol. 2023 Jan 18;13:1034350. doi: 10.3389/fneur.2022.1034350 (PMC9891292; doi:10.3389/fneur.2022.1034350)
Supplement: Supplementary file 1 [file Table_1.docx]

| **Table S1 Baseline and procedural characteristics** | | | | |
| --- | --- | --- | --- | --- |
| **Characteristics** | **Included patientI(n=177)** | **Excluded(n=224)** | **Test used** | ***P* value** |
| Age > 65 | 100(56.5%) | 134(59.8%) | χ2 | 0.503 |
| Femal | 52(29.4%) | 69(30.8%) | χ2 | 0.758 |
| NIHSS on admission | 3.48±3.84 | 3.53±3.88 | T test | 0.929 |
| Hypertension | 131(74.0%) | 148(66.1%) | χ2 | 0.086 |
| Diabetes mellitus | 71(40.1%) | 84(37.5%) | χ2 | 0.594 |
| Atrial fibrillation | 67(37.9%) | 65(29.0%) | χ2 | 0.062 |
